# Efficacy of electromechanical-assisted gait training on clinical walking function and gait symmetry after brain injury of stroke: a randomized controlled trial

**DOI:** 10.1038/s41598-022-10889-3

**Published:** 2022-04-27

**Authors:** Yeon Gyo Nam, Mun Jung Ko, Soo Kyung Bok, Nam-Jong Paik, Chi-Yeon Lim, Jin Won Lee, Bum Sun Kwon

**Affiliations:** 1 Institute of Posture Science, Dongguk University, Gyeongju, Republic of Korea; 2 Department of Biostatistics, School of Medicine, Dongguk University, Goyang, Republic of Korea; 3 Department of Rehabilitation Medicine, Chungnam National University College of Medicine, Chungnam, Republic of Korea; 4 Department of Rehabilitation Medicine, Seoul National University Bundang Hospital, Seoul, Republic of Korea; 5 HMH Co. Ltd, Incheon, Republic of Korea; 6 Department of Rehabilitation Medicine, Graduate School of Dongguk University, Seoul, Republic of Korea

**Keywords:** Neurological disorders, Therapeutics, Electronic and spintronic devices, Randomized controlled trials

## Abstract

Electromechanical-assisted gait training may be an effective intervention to promote motor recovery after brain injury. However, many studies still have difficulties in clarifying the difference between electromechanical-assisted gait training and conventional gait training. To evaluate the effectiveness of electromechanical-assisted gait training compared to that of conventional gait training on clinical walking function and gait symmetry of stroke patients. We randomly assigned patients with stroke (*n* = 144) to a control group (physical therapist-assisted gait training) and an experimental group (electromechanical gait training). Both types of gait training were done for 30 min each day, 5 days a week for 4 weeks. The primary endpoint was the change in functional ambulatory category (FAC). Secondary endpoints were clinical walking functions and gait symmetries of swing time and step length. All outcomes were measured at baseline (pre-intervention) and at 4 weeks after the baseline (post-intervention). FAC showed significant improvement after the intervention, as did clinical walking functions, in both groups. The step-length asymmetry improved in the control group, but that in the experimental group and the swing-time asymmetry in both groups did not show significant improvement. In the subgroup analysis of stroke duration of 90 days, FAC and clinical walking functions showed more significant improvement in the subacute group than in the chronic group. However, gait symmetries did not show any significant changes in either the subacute or the chronic group. Electromechanically assisted gait training by EXOWALK was as effective as conventional gait training with a physiotherapist. Although clinical walking function in the subacute group improved more than in the chronic group, gait asymmetry did not improve for either group after gait training.

**Trial registration**: KCT0003411 Clinical Research Information Service (CRIS), Republic of Korea.

## Introduction

In some people with disabilities, all or some motor functions of the lower limbs are significantly decreased because of brain lesions. Rehabilitation following brain injury from stroke can improve their walking efficiency and functional independence for activities of daily living^1^. For gait rehabilitation, clinicians prefer a repetitive approach with higher intensities of walking-practice programs^2^. Electromechanical-assisted gait training that requires repetitive tasks can improve the neuro-plasticity for motor learning with a focus on reorganization of brain tissue, resulting in better balance and a faster gait^3^.

In the 2020 Cochrane review, the people who received electromechanical-assisted gait training in combination with physiotherapy after stroke are more likely to achieve independent walking than people who receive gait training without these devices. Specifically, people in the first three months after stroke and those who are not able to walk seem to benefit most from this type of intervention^4^. However several studies reported that electromechanically assisted gait training can improve the gait function in patients with chronic stroke^5,6^. Accumulating evidence has suggested that high-intensity repetitive task-specific practice might be the most effective strategy for promoting motor recovery after a stroke^7^. Electromechanical-assisted gait training represents such a treatment option^8^.

Improvement of gait symmetry was achieved electromechanical gait training and related to impairment in balance^9^. According to a recent pilot study, the gait symmetry of patients with stroke came closer to the normal range after gait training with a Wearable Hip-Assist device for 4 weeks^10^. A recent review has indicated that there is still a need for well-designed, large-scale, multicenter studies to evaluate the benefits of electromechanical-assisted gait training for walking after stroke^4^.

**Figure 1:**
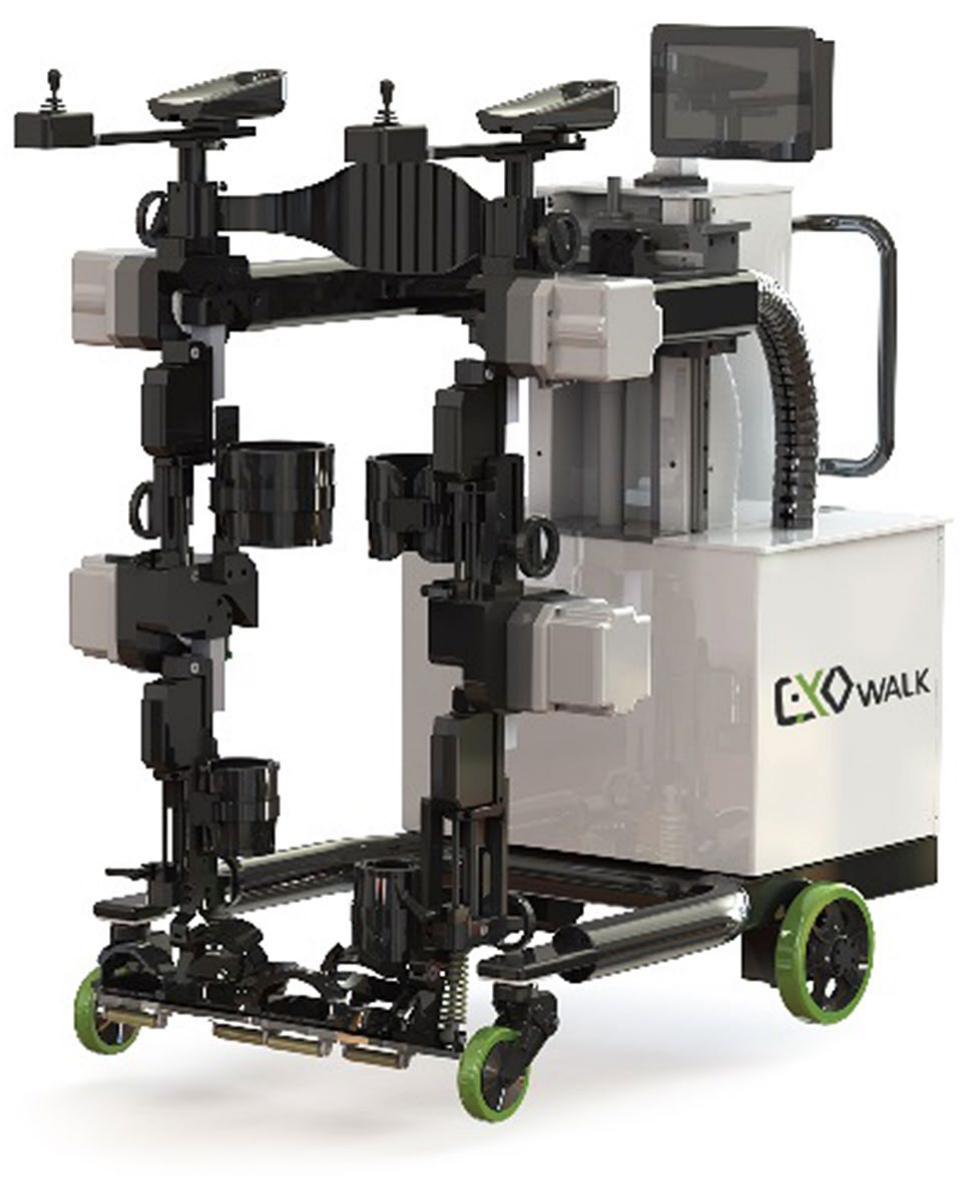
EXOWALK (HMH Co. Ltd, South Korea).

EXOWALK (HMH Co., HR-01, A67020.02, Grade2, South Korea) is an electromechanically assisted gait trainer that can provide stable and firm standing ability with little chance of falling (Fig. 1). It obviates the need for an additional cane or walker more than do currently popular exoskeletons. Such designs are user-friendly without needing a harness for weight support.

Many studies have investigated the effectiveness by clinical evaluation and still have found it difficult to clarify the difference between conventional and electromechanically assisted gait training^11,12^, sometimes because of having too few subjects^13–15^. In systematic reviews of electromechanically assisted gait training plus physiotherapy versus physiotherapy alone, there is still a need for large-scale and multicenter studies with good design after strokes^4^. Our purpose in this prospective study was to use a multi-center randomized design to investigate the effect of electromechanically assisted gait training using the EXOWALK, which provides repetitive training with normal symmetric gait on clinical walking function and gait symmetry of stroke patients.

## Methods

This was a multicenter, randomized, prospective, and parallel-group study on the efficacy and safety of the electromechanical gait trainer EXOWALK. All enrolled subjects were patients with stroke. The following three clinical research centers participated in this trial: Dongguk University Ilsan Hospital, Chungnam National University Hospital, and Seoul National University Bundang Hospital. This research protocol was approved by each hospital as follows: Dongguk University Ilsan Hospital’s Institutional Review board (IRB No. DUIH 2018-08-026-001), Chungnam National University Hospital’s IRB (IRB No. CNUH 2018-09-033) and Seoul National University Bundang Hospital’s IRB (IRB No. B-1810/497-001). This study was registered at the Clinical Research Information Service (CRIS, KCT0003411; date of registration, 03/01/2019). We obtained informed consent from all participants and did all the research in accordance with the Declaration of Helsinki.

Given the data of patients who agreed to participate in this study, we screened to select eligible subjects based on the following inclusion and exclusion criteria. Inclusion criteria were:

1. those who had had a stroke,
2. those who had a score of 10 or more for the Mini-Mental State Examination (MMSE),
3. those who had a Modified Ashworth Scale (MAS) Grade 2 or lower, and
4. those who could stand alone.

Exclusion criteria were:

1. those with poor cognition that made it difficult for them to carry out instructions,
2. those with ataxia that caused unstable standing balance,
3. those with spasticity MAS Grade 3 or above,
4. those with severe leg arthritis, and
5. those with difficulty walking because of joint problems of the lower leg.

This was a randomized, controlled, single-blind trial. We assigned subjects into an experimental group or a control group by using a randomized allocation table for subjects who met the inclusion/exclusion criteria and who agreed to participate in this study. Randomization tables were created for each research organization. Randomization was done using a random-number generator computerized with a block randomization method in SAS version 9.4 (SAS institute, Inc., Cary, NC, USA). Outcome assessors were blinded for reducing the bias. Intervention and evaluation were done by different physiotherapists with five years or more of experience, to increase the reliability by minimizing the measurement error. At enrollment, we instructed patients not to reveal their allocation arm to the outcome assessor. The researcher who did the randomization and data analyses was not involved in any assessment or training.

We prospectively enrolled 144 suitable patients from November 2018 to August 2020; each hospital registered 48 subjects. We assigned subjects into an experimental group or a control group. Patients in both groups were given 30 min of training per session, five times per week for 4 weeks. In addition, we did basic rehabilitation (neurodevelopmental treatment, exercise for range of motion and strengthening) for both groups. The experimental group received electromechanically assisted gait training with EXOWALK and the control group received conventional gait rehabilitation treatment by therapists. We recommended the patients in this study to receive the electromechanical exoskeleton-assisted gait training at a comfortable speed. Although the maximum safe velocity of this device was 2.3 km/h, we provided the gait training velocity under 1.8 km/h according to the initial evaluation to prevent fatigue. For subjects in the control group, the physiotherapist guided and walked the patient while assisting the subject on the side or the back.

### Outcome measures

In this study, we measured and documented the demographic and clinical characteristics of subjects after screening. Demographic information included sex, date of birth, height, weight, and joint problems (or not). Clinical characteristics included the name of the diagnosis, the cause of the disability (brain infarction, cerebral hemorrhage), the paralyzed side (right, left), whether they could express intention (standard: MMSE 10 or higher), and the lower-limb spasticity score (standard: MAS grade 2 or lower).

The change in functional ambulatory category (FAC)^16^ from before to after gait training was the primary endpoint for evaluating the efficacy of electromechanical exoskeleton-assisted gait training. We evaluated the FAC at baseline (pre-intervention) and at 4 weeks after the baseline (post-intervention), by dividing the degree of need for assistance when walking from 1 to 6. FAC level ranged from Level 1 (‘nonfunctional’) to Level 6 (‘independent without help for non-level surfaces’).

The second endpoint had seven assessments. First, we used the RMI^17^ to evaluate motor skills. It consisted of 15 questions step by step, depending on the level, ranging from bed rotation to running. Each of the 15 questions scored 1 point (if yes) or 0 (if no). The sum was used as a result of the evaluation.

Second, we used walking velocity in 10 mWT^18^ to measure the speed during a 10-m walk. The unit was m/s (meter per second). Similarly, we evaluated walking capacity with a 6 mWT^19^ to measure the distance that one could walk for 6 min. The unit was m (meter).

The fourth item was MI^20^, which we evaluated as 1 to 99 points by measuring the lower-leg force level from the ankle to the knee. Assessment items consisted of three questions, each with a score of 0/9/14/19/25/33. The sum of scores was used as the result of the evaluation.

The fifth item was BBS^21^ to evaluate the balance ability with 0 to 56 points. Each of the 14 questions was scored 0 to 4 points. The sum of scores was used as the result of the evaluation.

The last two evaluation items were quantitative gait symmetry of swing time and step length measured by a dynamic foot-pressure device (HumanTrack; Rbiotech Co., Ltd., Seoul, Korea)^22^. Swing time was defined as the time obtained by subtracting previous toe-off time from heel-strike time. Step length is the direct distance between the point of initial contact of one foot and the point of initial contact of the opposite foot. We calculated the following ratios for gait symmetry^23^.

Swing-time symmetry = paretic swing time/nonparetic swing time.

Step-length symmetry = nonparetic step length/paretic step length.

For the patients who consented to additional evaluation, we did all measurements 4 weeks after the last intervention (follow-up). Physical content of clinical alteration was reported by auditors, practitioners, and patients at each visit. All indications, data of onset, and period were recorded.

### Analysis

In a previous trial^24^, the mean change of FAC between pre- and post-intervention was 0.54 in the control group (conventional gait training with physiotherapist) and 1 in the test group. We expected the medical devices used in this work to achieve approximately 25% better performance, because we provided a longer intervention time than did the reference, and we assumed the change of the mean value to be 1.25. Thus, we assumed that the difference in change between the test medical device and the control (conventional gait training) group was 0.71, and that the largest value, 1.4, was needed for conservative access to the standard deviation. Thus, 65 participants were needed for each group to achieve 80% power at a significant level of 0.05. Considering a possible dropout rate of 10%, we chose the sample size as 144 (72 participants per group).

For demographic and clinical characteristics, categorical variables such as sex, joint problems, disability cause, paralyzed side, and lower extremity MAS scores are presented as frequency and percentage. They were analyzed for pre-homogeneity with chi-squired tests. Continuous variables are presented as mean, standard deviation (SD), and range of minimum and maximum (Min, Max). For height and weight satisfying normality, we analyzed pre-homogeneity using a Student's *t-*test. For age not satisfying normality, we used a Wilcoxon rank sum test.

All values of primary and secondary endpoints are presented as mean and SD (Mean ± SD). Although basic results of FAC were scored 1, 2, 3, 4, 5, and 6 on an ordinal scale, FAC is presented as mean and SD, and was one of the most popular tools for measuring ambulatory function^25,26^. Within each group, we analyzed the changes in the values of pre-intervention and post-intervention using a paired *t*-test if normality was satisfied or a Wilcoxon's signed rank test if not satisfied. In addition, for comparison between pre-intervention and post-intervention values of the test and control groups, we used a Student's *t-*test and a Wilcoxon's rank sum test (Table 2).

Stroke duration was the most important factor that affected results. Subgroup analysis compared variations between subjects with stroke durations of 90 days or less and those with 91 days or more (over 91 days) in the experimental group. For all results, we analyzed values of pre-intervention and post-intervention changes using a paired *t*-test if normality was satisfied and a Wilcoxon's signed rank test if normality was not. In order to compare pre-post changes between groups, we analyzed them by using a Student's *t*-test or a Wilcoxon's rank sum test, depending on whether normality was satisfied (Tables 3, 4). We did all statistical analyses using SAS version 9.4 or later. All statistical tests were two-sided, and the level of significance was set at *p* < 0.05.

### Ethics approval and consent to participate

This research protocol was approved by the Institutional Review Board (IRB) of Dongguk University Ilsan Hospital, Chungnam National University Hospital, and Seoul National University Bundang Hospital. This study was performed following protocols approved by the IRB and included only patients who provided written informed consent.

## Results

**Figure 2:**
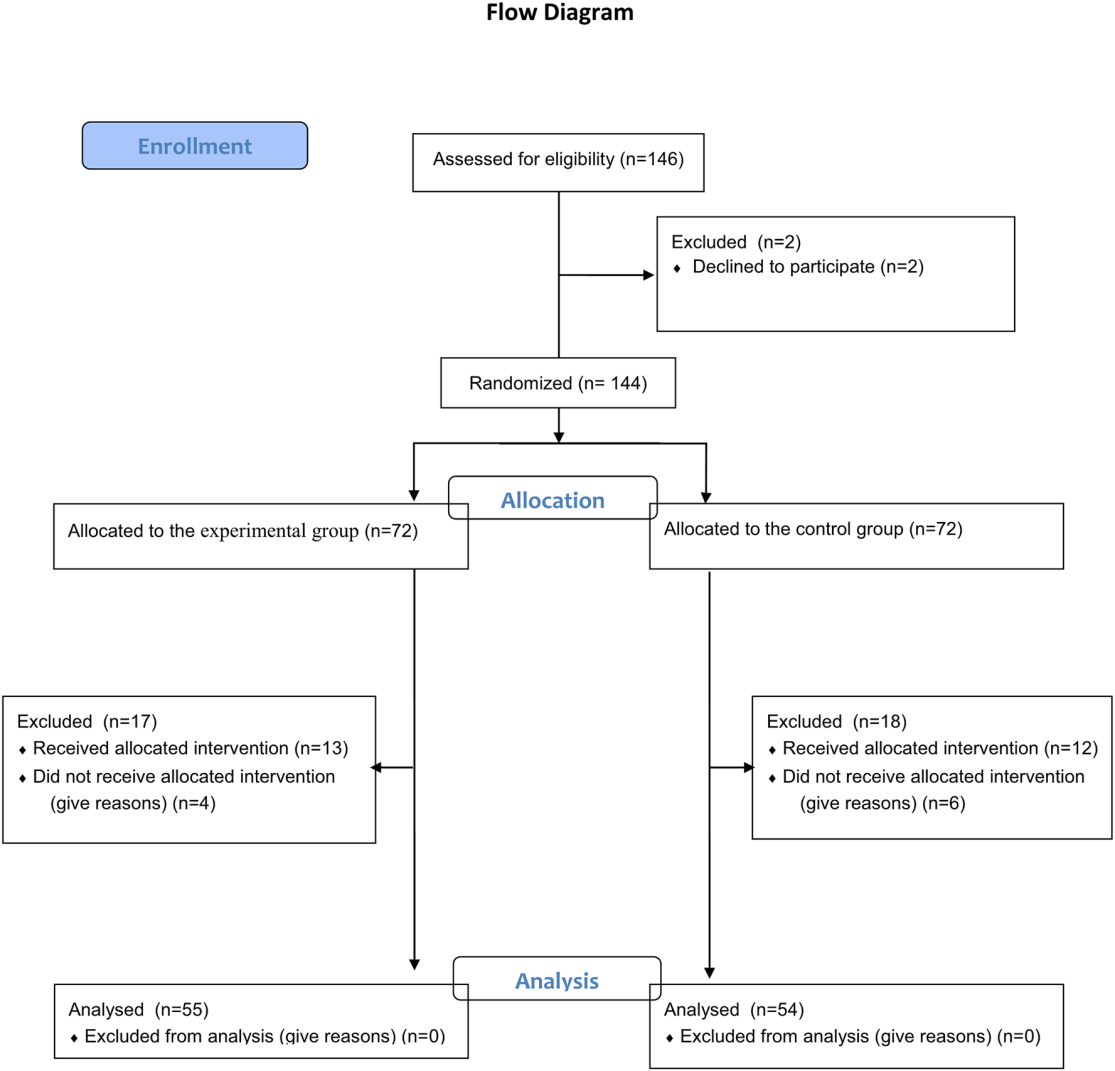
CONSORT flow diagram.

We included 144 subjects in this study. Of them, 104 completed gait training and outcome measures at 4 weeks after initiation of the intervention. In the experimental group, withdrawal before first evaluation for personal reasons (n = 2), withdrawal before the first treatment for personal reasons (*n* = 2), withdrawal after the first treatment for personal reasons (*n* = 4), and incomplete second evaluation (*n* = 9) occurred (Fig. 2). In the control group, there was withdrawal before first evaluation for personal reasons (*n* = 4), withdrawal before the first treatment for personal reasons (*n* = 2), withdrawal after the first treatment for personal reasons (*n* = 7), and incomplete second evaluation (*n* = 5) (Fig. 2). There were no significant differences in baseline characteristics between the control and experimental groups. All subjects could control their gait direction and speed. The mean MMSE was 24.81 ± 4.65 in the experimental group and 23.69 ± 5.17 in the control group. All subjects could ambulate with or without the assistance of another person (Table 1).

**Table 1:** The baseline characteristic of the experimental and control group.

Variables	Control group n = 55	Experimental group n = 54	p-value
**Sex, n (%)**			
Male	35 (63.64%)	34 (62.96%)	0.942*
Female	20 (36.36%)	20 (37.04%)	
**Age**			
Mean ± SD	62.42 ± 15.04	60.63 ± 15.61	0.728##
Range(Min, Max)	22, 89	23, 86	
**Height, cm**			
Mean ± SD	163.23 ± 9.70	164.07 ± 7.12	0.605#
Range(Min, Max)	146.4, 185.0	148.0, 177.8	
**Weight, kg**			
Mean ± SD	64.24 ± 11.21	64.22 ± 10.70	0.990#
Range(Min, Max)	39.4, 95.1	38.7, 98.1	
**Type, n(%)**			
Infarction	34 (61.82%)	33 (61.11%)	0.939*
Hemorrhage	21 (38.18%)	21 (38.89%)	
**Paretic side, n (%)**			
Rt	28 (50.91%)	25 (46.3%)	0.630*
Lt	27 (49.09%)	29 (53.70%)	
**MMSE**			
Mean ± SD	23.69 ± 5.17	24.81 ± 4.65	0.3063##
Range(Min, Max)	13, 30	10, 30	
**MAS**			
0	43 (78.18%)	43 (79.63%)	0.9362*
1	8 (14.55%)	6 (11.11%)	
1.5	1 (1.82%)	1 (1.85%)	
2	3 (5.45%)	4 (7.41%)	
**Onset duration, day**			
Mean ± SD	522.40 ± 1220.70	767.17 ± 1435.78	0.1139##
Range(Min, Max)	3, 7529	1, 8435	

*MMSE* Mini-mental state examination, *MAS* Modified Ashworth Scale.

*p-value obtained from Chi-square test.

^#^p-value obtained from Student's t-test.

^##^p-value obtained from Wilcoxon rank sum test.

The mean FAC in the experimental group was 3.15 ± 1.39 before intervention (pre-intervention) and 4.22 ± 1.37 after the intervention (post-intervention) (Table 2). The mean FAC in the control group was 3.11 ± 1.29 pre-intervention and 4.20 ± 1.03 post-intervention. Between pre-intervention and post-intervention, the change in FAC showed significant improvement in both groups. However, the change in FAC did not differ between the two groups (Table 2). In the experimental group, the outcomes of clinical walking functions showed improvement after intervention, but those of quantitative gait symmetry did not. In the control group, the outcome of clinical waking functions and step-length asymmetry showed improvement, but that of swing-time asymmetry did not. However, the change of all secondary outcomes between two groups was not different (Table 2).

**Table 2:** The difference of outcome changes in the experimental and control group.

Variables	Control group					Experimental group					95% Confidence Limits(CL)†	p-value between groups*
	N	Pre	Post	Difference	p-value	N	Pre	Post	Difference	p-value		
FAC	55	3.11 ± 1.29	4.2 ± 1.03	1.09 ± 1.01	< 0.0001##	54	3.15 ± 1.39	4.22 ± 1.37	1.07 ± 0.82	< 0.0001##	− 0.000007, 0.000017	0.934
RMI	55	6.51 ± 3.82	8.56 ± 3.68	2.055 ± 3.21	< 0.0001##	54	6.69 ± 3.42	8.31 ± 3.99	1.63 ± 2.52	< 0.0001##	− 1.000037, 0.000034	0.495
10mWT (m/s)	55	0.45 ± 0.29	0.57 ± 0.33	0.17 ± 0.23	< 0.0001##	54	0.50 ± 0.52	0.58 ± 0.55	0.12 ± 0.55	< 0.0001##	− 0.11 , 0.02	0.175
6MWT	55	131.09 ± 101.40	184.58 ± 123.83	55.30 ± 106.98	< 0.0001##	54	115.95 ± 105.03	180.93 ± 127.58	61.48 ± 91.08	< 0.0001##	− 21 , 30	0.892
MI	55	55.24 ± 16.48	66.69 ± 17.23	11.45 ± 13.87	< 0.0001##	54	50.07 ± 19.78	61.56 ± 20.42	11.19 ± 12.79	< 0.0001##	− 5 , 5	0.835
BBS	55	26.22 ± 17.17	38.67 ± 13.48	12.45 ± 13.91	< 0.0001##	54	26.33 ± 17.23	37.13 ± 15.30	10.80 ± 11.92	< 0.0001##	− 3 , 3	0.832
Swing Time Symmetry	19	3.737 ± 8.753	1.782 ± 1.196	− 1.955 ± 8.846	1.000##	16	1.555 ± 0.883	1.261 ± 0.502	− 0.293 ± 1.02	0.4256##	− 0.899 , 0.503	0.4813
Step length Symmetry	19	0.768 ± 0.332	0.905 ± 0.32	0.137 ± 0.348	0.0494##	16	0.829 ± 0.337	0.889 ± 0.374	0.06 ± 0.286	0.8603##	− 0.303 , 0.078	0.1784

*FAC* functional ambulation categories, *RMI* rivermead mobility index, *10mWT* 10-meter walk test, *6MWT* 6-minute walk test, *MI* motricity index, *BBS* berg balance scale.

^#^p-value obtained from Paired t-test.

^##^p-value obtained from Wilcoxon signed rank test.

^†^CL obtained from Wilcoxon's rank sum test.

*p-value obtained from Wilcoxon rank sum test.

When each group was divided into two groups according to stroke duration of 90 days, the changes in clinical walking functions were greater in the below-90-days group than in the over-91-days group in both the control group (Table 3) and the experimental group (Table 4). However, changes in gait symmetries did not show any significant difference between the two groups (Tables 3, 4). Adverse events were not found during gait training in either group.

**Table 3:** The difference of outcome changes with stroke duration in the control group.

Variables	Stroke duration ≤ 90 days					Stroke duration ≥ 91 days					95% Confidence Limits(CL)†	p-value between groups*
	N	Pre	Post	Difference	p-value	N	Pre	Post	Difference	p-value		
FAC	31	2.94 ± 1.18	4.35 ± 0.91	1.42 ± 0.99	< 0.0001##	24	3.33 ± 1.40	4.00 ± 1.14	0.67 ± 0.87	< 0.0001##	0.000033, 1.000069	0.0054
RMI	31	6.35 ± 3.94	9.06 ± 3.70	2.71 ± 3.80	0.0004#	24	6.71 ± 3.75	7.92 ± 3.62	1.21 ± 2.00	0.007#	0.000071, 2.999938	0.0211
10mWT	31	0.37 ± 0.30	0.61 ± 0.34	0.24 ± 0.20	< 0.0001##	24	0.38 ± 0.35	0.53 ± 0.31	0.15 ± 0.29	0.0011##	0.0002 , 0.21	0.0471
6MWT	31	132.61 ± 110.32	204.39 ± 119.48	71.77 ± 85.11	0.0011##	24	107.27 ± 94.17	159.00 ± 127.16	51.73 ± 143.37	0.0233##	12 , 87	0.0164
MI	31	55.65 ± 16.41	72.97 ± 15.96	17.32 ± 13.74	< 0.0001#	24	54.71 ± 16.92	58.58 ± 15.59	3.88 ± 9.97	0.0694#	6 , 19	0.0005
BBS	31	24.52 ± 17.67	39.13 ± 13.69	14.61 ± 13.78	< 0.0001##	24	28.42 ± 16.63	38.08 ± 13.48	9.67 ± 13.87	< 0.0001##	− 1 , 10	0.1141
Swing Time Symmetry	9	1.924 ± 1.58	1.692 ± 1.243	− 0.232 ± 1.764	1.000##	10	5.369 ± 12.032	1.864 ± 1.213	− 3.505 ± 12.17	0.9219##	− 1.482 , 1.544	0.9679
Step length Symmetry	9	0.714 ± 0.243	0.921 ± 0.218	0.208 ± 0.381	0.1403#	10	0.817 ± 0.403	0.89 ± 0.403	0.073 ± 0.322	0.4916#	− 0.238 , 0.517	0.9679

*FAC* functional ambulation categories, *RMI* rivermead mobility index, *10mWT* 10-meter walk test, *6MWT* 6-minute walk test, *MI* motricity index, *BBS* Berg balance scale.

^#^p-value obtained from Paired t-test.

^##^p-value obtained from Wilcoxon signed rank test.

^†^CL obtained from Wilcoxon's rank sum test.

*p-value obtained from Wilcoxon rank sum test.

**Table 4:** The difference of outcome changes with stroke duration in the experimental group.

Variables	Stroke duration ≤ 90 days					Stroke duration ≥ 91 days					95% Confidence Limits(CL)†	p-value between groups*
	N	Pre	Post	Difference	p-value	N	Pre	Post	Difference	p-value		
FAC	21	2.95 ± 1.16	4.52 ± 1.25	1.57 ± 1.08	< 0.0001##	33	3.27 ± 1.53	4.03 ± 1.42	0.76 ± 0.79	< 0.0001##	0.000015, 1.000036	0.006
RMI	21	6.43 ± 4.24	9.19 ± 4.76	2.76 ± 3.45	0.0013##	33	6.85 ± 3.03	7.76 ± 3.38	0.91 ± 1.31	0.0003##	0.000038, 2.999967	0.0214
10mWT	21	0.38 ± 0.28	0.70 ± 0.67	0.33 ± 0.51	< 0.0001##	33	0.50 ± 0.63	0.49 ± 0.45	− 0.01 ± 0.51	0.0115##	0.0561 , 0.272	0.0053
6MWT	21	126.19 ± 85.71	205.14 ± 115.51	78.95 ± 94.75	0.0011#	33	98.89 ± 116.34	160.05 ± 135.28	61.15 ± 115.16	0.0001##	− 16 , 70	0.2008
MI	21	51.14 ± 16.44	70.10 ± 18.66	18.95 ± 13.61	< 0.0001#	33	49.39 ± 21.86	55.64 ± 19.73	6.24 ± 9.50	0.0007#	5 , 22	0.0012
BBS	21	23.38 ± 17.65	39.71 ± 14.29	16.33 ± 12.76	< 0.0001##	33	28.21 ± 16.96	35.48 ± 15.91	7.27 ± 10.03	< 0.0001##	3 , 13	0.0014
Swing Time Symmetry	8	1.471 ± 1.126	1.104 ± 0.396	− 0.366 ± 1.403	0.8438##	8	1.639 ± 0.623	1.418 ± 0.572	− 0.22 ± 0.5	0.253#	− 0.447 , 0.756	0.4428
Step length Symmetry	8	0.942 ± 0.251	1.065 ± 0.296	0.123 ± 0.366	0.8438##	8	0.717 ± 0.389	0.714 ± 0.378	− 0.003 ± 0.181	0.9659#	− 0.218 , 0.460	0.7183

*FAC* functional ambulation categories, *RMI* Rivermead Mobility Index, *10mWT* 10-meter walk test, *6MWT* 6-minute walk test, *MI* motricity index, *BBS* Berg balance scale.

^#^p-value obtained from Paired t-test.

^##^p-value obtained from Wilcoxon signed rank test.

^†^CL obtained from Wilcoxon's rank sum test.

*p-value obtained from Wilcoxon rank sum test.

## Discussion

Electromechanical gait-training devices have been developed; their effectiveness has been proven by clinical studies for stroke patients^27^. Gait rehabilitations with an electromechanical gait-training device can increase the length, intensity, and number of physiotherapy sessions^28^. For clinical effects of electromechanical-assisted gait training, Mehrholz et al. have demonstrated that it could improve post-stroke independent walking recovery when it is combined with physical therapy in patients suffering from a stroke^4^. It is effective for patients in the first three months after stroke and for those who are not able to walk^5^. In fact, there is growing evidence that the motor system is plastic following a stroke and that motor training can aid patients, particularly in the first 3 months^29–31^.

EXOWALK is an electromechanical exoskeleton-assisted gait-training device. It has a unique design that applies an exoskeleton in front of a robotic body and makes walking possible using a motorized wheel controlled by the patient. We used two randomized controlled trials (RCTs) to investigate the effect of electromechanically assisted gait training with EXOWALK on stroke patients^31,32^. The first study revealed that gait training for 30 min with EXOWALK was effective^31^. The stroke patients had confidence in their gait and desire to continue gait training. However, the effect declined with increasing stroke duration^31^. We found that, for them, electromechanically assisted gait training with EXOWALK was not superior to conventional physiotherapy^32^. We also found that electromechanically assisted gait training was as effective as conventional gait training, although its effect was better for those with less than 90 days of stroke duration. These results were the same as our previous studies^33^.

The difference in FAC change between pre-intervention and post-intervention was 1.09 ± 1.01 in the control group and 1.07 ± 0.82 in the experimental group. When we did various interventions of gait training including conventional treatments for stroke patients, FAC showed an improvement in a range of 0.3 to 1.0^34–36^. In this study, changes in FAC and secondary outcomes were significant enough clinically. However, they declined in the over-91-days group. The Cochrane review by Mehrholz et al. revealed that electromechanically assisted training for walking after stroke did not improve the walking capacity or velocity^4^. In this study, walking capacity and velocity were improved after intervention in the below-90-days group. These were not related to walking symmetry.

**Figure 3:**
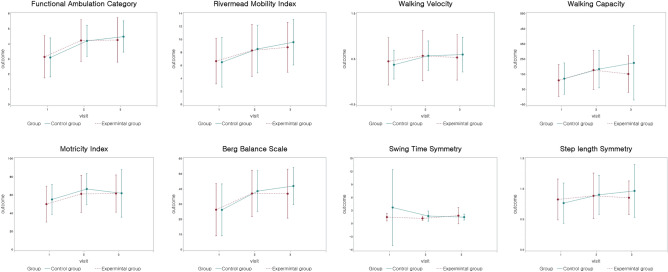
The changes of outcome measures pre-intervention (0 week), immediate post-intervention (4 weeks) and 4 weeks after intervention (follow up) of the control group (solid line) and experimental group (dotted line).

Electromechanically assisted gait training is effective in patients with acute and sub-acute stroke, but not in those with chronic stroke, according to subgroup analysis of 461 participants in the chronic phase, defined as more than 90 days after stroke^4^. When the experimental group was divided into two groups in terms of stroke duration of 90 days, most outcomes of subacute patients showed improvements more than did those of chronic patients in both control and experimental groups. In this study, 87 participants among 104 total patients underwent the follow-up evaluation. Baseline characteristics of follow-up evaluation showed no significant differences between the control group and the experimental group. Most outcomes showed significant improvements that were maintained at follow-up evaluation (Fig. 3).

Gait asymmetry and different values of gait parameters were additional gait characteristics of fallers^37^. When the lower extremities have different levels of ability, gait asymmetry might be an element for improving the dynamic balance^38^. We found that the changes in gait symmetries did not show significant improvement after electromechanically assisted gait training intervention for 4 weeks. The balance improved clinically, which might be related to muscle power but not to gait asymmetry, as shown by a previous RCT that investigated the effects of electromechanically assisted gait training on step-length symmetry in subacute stroke patients with hemiplegia and showed no significant difference between the control (gait training with the physiotherapy) group and the experimental group (electromechanically assisted gait training)^9^. Asymmetry of gait is related to increases in energy expenditure, reduced balance control, and risk of unaffected limb injury^39^. As motor impairment occurs over time, there is an adapted gait pattern^40^. Electromechanical gait training can provide much repetitive training with a normal gait pattern. However, it cannot change gait asymmetry in both subacute and chronic patients.

### Limitation

Since the inclusion criteria were for stroke patients who could stand alone, many patients who needed walking aids or assistance were registered. We did not evaluate their gait symmetry at the time of pre-intervention. Even though some of them could walk independently without walking aids or assistance, their measurements of gait symmetry post-intervention were not included in the result. Some old and chronic stroke patients had fixed deformity, and gait analysis was not relevant. Thus, there were few subjects with gait symmetry and more dropout patients than we expected; so the power of the result was small. We needed to estimate sample size more conservatively.

## Conclusions

The clinical walking function was improved significantly after electromechanically assisted gait training, and the improvement was the same as with conventional gait training with a physiotherapist. The clinical walking function of subacute patients after 4 weeks of gait training was improved more than that of chronic patients. However, the gait asymmetry was not improved in either subacute or chronic stroke patients.

## Data Availability

The datasets generated and/or analyzed during the current study are available from the corresponding author upon reasonable request.
